# Leukocyte Lysis and Cytokine Induction by the Human Sexually Transmitted Parasite *Trichomonas vaginalis*

**DOI:** 10.1371/journal.pntd.0004913

**Published:** 2016-08-16

**Authors:** Frances Mercer, Fitz Gerald I. Diala, Yi-Pei Chen, Brenda M. Molgora, Shek Hang Ng, Patricia J. Johnson

**Affiliations:** 1 Department of Microbiology, Immunology & Molecular Genetics, University of California, Los Angeles, Los Angeles, California, United States of America; 2 Molecular Biology Institute, University of California, Los Angeles, Los Angeles, California, United States of America; New York University, UNITED STATES

## Abstract

*Trichomonas vaginalis (Tv)* is an extracellular protozoan parasite that causes the most common non-viral sexually transmitted infection: trichomoniasis. While acute symptoms in women may include vaginitis, infections are often asymptomatic, but can persist and are associated with medical complications including increased HIV susceptibility, infertility, pre-term labor, and higher incidence of cervical cancer. Heightened inflammation resulting from *Tv* infection could account for these complications. Effective cellular immune responses to *Tv* have not been characterized, and re-infection is common, suggesting a dysfunctional adaptive immune response. Using primary human leukocyte components, we have established an *in vitro* co-culture system to assess the interaction between *Tv* and the cells of the human immune system. We determined that *in vitro*, *Tv* is able to lyse T-cells and B-cells, showing a preference for B-cells. We also found that *Tv* lysis of lymphocytes was mediated by contact-dependent and soluble factors. *Tv* lysis of monocytes is far less efficient, and almost entirely contact-dependent. Interestingly, a common symbiont of *Tv*, *Mycoplasma hominis*, did not affect cytolytic activity of the parasite, but had a major impact on cytokine responses. *M*. *hominis* enabled more diverse inflammatory cytokine secretion in response to *Tv* and, of the cytokines tested, *Tv* strains cleared of *M*. *hominis* induced only IL-8 secretion from monocytes. The quality of the adaptive immune response to *Tv* is therefore likely influenced by *Tv* symbionts, commensals, and concomitant infections, and may be further complicated by direct parasite lysis of effector immune cells.

## Author Summary

The unicellular parasite *Trichomonas vaginalis* (*Tv*) causes the most common non-viral sexually transmitted infection worldwide, with approximately one quarter of a billion people infected annually. *Tv* infections are linked to pre-term and low-weight infant birth, increased susceptibility to and transmission of HIV infection, and increased aggressiveness of urogenital tract cancers. How the immune system responds to *Tv*, and how the parasite persists in the presence of the immune system, is not well understood. We show that *Tv* can kill human immune cells. We found that a clinical isolate of *Tv* is more efficient at killing immune cells than a laboratory-adapted strain, and that *Tv* preferentially targets B-cells. This killing activity is mediated by both contact-dependent and soluble factors. Killing immune cells could allow *Tv* to subvert or disable the immune system. Conversely, we found that cells of the immune system respond to *Tv* by secreting soluble inflammatory mediators called cytokines, mainly interleukin-8. However, these cells secrete greater amounts, and a broader profile of inflammatory cytokines when they encounter *Tv* harboring its symbiont bacterium, *Mycoplasma hominis*. We conclude that *Tv* may interact with immune cells in different ways, depending on the strain of *Tv* and the symbiont it harbors.

## Introduction

*Trichomonas vaginalis* (*Tv*) is a unicellular, aerotolerant, flagellated protozoan parasite that is an obligate extracellular pathogen, restricted to humans [1]. *Tv* adheres to and lyses host epithelial cells [2], followed by phagocytosis of cellular contents [3]. *Tv* destruction of the epithelial layer in the female reproductive tract (FRT) is apparent in the clinical presentation of “strawberry cervix,” in which red lesions are visible on the external surface of the cervix of infected women [1]. Once thought to be a commensal member of the vaginal microflora, *Tv* is now known to be pathogenic and is responsible for the most common non-viral sexually transmitted infection (STI) in the United States and worldwide: trichomoniasis [4]. The WHO reports ~275 million cases annually [1], but this number is likely a gross underestimation, as the CDC estimates that at least 50% percent of cases are asymptomatic. In the United States, an estimated 8–10 million new infections occur annually [5]. Alarmingly, trichomoniasis is on the rise in adolescents [6], and in dense urban areas prevalence can be as high as 50% [7]. Because *Tv* responds well to metronidazole, an antibiotic that specifically kills anaerobic cells [1], most symptomatic cases are successfully treated. However, emergence of metronidazole-resistant strains continues to increase [4,8–12], and metronidazole treatment may not efficacious for preventing pregnancy-related *Tv* complications [13,14]. Moreover, the association of subclinical infection with complications affecting women’s reproductive health [6,15] necessitates a better understanding of how *Tv* causes disease, and how the immune system responds to the parasite.

Trichomoniasis is associated with increased susceptibility to HIV, HSV-2, pelvic inflammatory disorder, pre-term and low-weight infant birth, infertility, and endometritis [15]. *Tv* infection is also associated with bacterial vaginosis, suggesting a disruption of the microflora [6]. In addition, *Tv* infection or serostatus has been correlated with an increased incidence of cervical cancer [16,17], especially invasive types [18–20]. Although *Tv* is typically asymptomatic in men [21], they are commonly infected and trichomonads can be detected in prostate tissue [22]. *Tv* infection in men has been linked to invasive forms of prostate cancer [23], and infertility [24]. It is thought that *Tv*- induced inflammation can exacerbate existing neoplastic lesions, increasing chances of malignancy [25,26].

*Tv* often co-exists with a symbiont bacterium, *Mycoplasma hominis* [27]. Otherwise axenic cultures of clinical isolates and lab strains of *Tv* often contain *M*. *hominis*, growing both externally to and within *Tv* [28,29]. This is unlikely to be an artifact of laboratory-introduced contamination, as a recent study of clinical isolates found the prevalence of *M*. *hominis* in *Tv* isolates to be 56% [30]. The prevalence of the biological association of *Tv* and *M*. *hominis* underscores the epidemiological relevance of studying *Tv* strains containing *M*. *hominis*.

Despite the association of *Tv* with numerous complications of a putative inflammatory etiology, how the immune system responds to and clears *Tv* is not well understood. Neutrophils are usually abundant during acute infection [31,32]. Antibodies can be detected in sera of infected persons [1,33,34], and after experimental challenge in a mouse model [35]. Peripheral blood mononuclear cells (PBMC) from infected persons proliferate *in vitro* in response to re-call antigen [36], and CD4+T cells were shown to be present after experimental mouse infection [35]. However, partner re-infection is common [1], indicating that there may be inadequate formation of immunological memory, or that *Tv* subverts effective adaptive immune responses. *Tv* has also been shown to induce IL-8 secretion from primary human monocytes [37]. In addition, the *Tv* symbiont *Mycoplasma hominis* has been shown to enable induction of an array of inflammatory cytokines in a macrophage cell line following *Tv* encounter [38]. However, studies examining the cytokine profile following *Tv* encounter with primary human monocytes have yet to be performed.

*Tv* shares a niche with numerous other commensal microorganisms in the FRT [39], and infection is often concomitant with other STIs [1,5]. Leukocytes populate mucosal tissues of the FRT [40], where they manage responses to commensals and other STIs. Leukocytes are predominant in the lamina propria, but may also follow trans-epithelial chemokine signals to home to the luminal side of the mucosa [41]. *Tv* may therefore encounter leukocytes while adhering to epithelial cells on the luminal side of the FRT, or approaching the lamina propria as the epithelial layer is breached by *Tv* cytolysis of epithelial cells. *Tv* has been demonstrated to phagocytose human leukocytes [42], indicating that direct leukocyte killing could contribute to *Tv* immune subversion; however, the efficiency, kinetics, and cell-specificity of this process is unknown.

Characterizing the type of immune response that *Tv* stimulates and determining whether *Tv* cytotoxic activity can kill leukocytes will be important to understanding why *Tv* is often persistent, how *Tv* infection may lead to inflammatory sequelae, and how *Tv* infection may affect the microbiome in the FRT. *Tv* is a human-specific pathogen, and most attempts at experimental mouse infection have failed to sustain adequate parasite titers, with the exception of a model using pre-treatment with estrogen and dexamethasone [43], both of which are immunosuppressive and therefore undesirable for analysis of immune function. In addition, specific host-surface proteins are implicated in pathogenesis [44], so using cells of the natural host for these studies is optimal. Using primary human leukocytes, here we show that *Tv* is able to kill immune effector cells, showing a preference for B-cells, and that cytokine responses induced by the parasite are largely dependent on the symbiont *M*. *hominis*.

## Methods

### Ethics statement

All work with cells from human blood donation was done in compliance with the UCLA School of Medicine IRB Committee.

### *Trichomonas vaginalis* strains and culture

*Tv* strains G3 (Beckenham, UK 1973, ATCC-PRA-98), and MSA1132 (Mt. Dora, Fla, USA 2008) were grown in TYM medium supplemented with 10% horse serum (Sigma), 10 U/ml penicillin (Invitrogen), 10 μg/ml streptomycin (Invitrogen), 180 μM ferrous ammonium sulfate, and 28 μM sulfosalicylic acid [45] at 37°C. Strains were passaged daily and maintained at an approximate concentration of 1 x 10^5^–2 x 10^6^ cells/ mL. To generate *M*. *hominis* free strains, *Tv* was grown in the presence of 50 μg/ml chloramphenicol and 5 μg/ml tetracycline (Sigma-Aldrich), supplemented daily for at least 5 days and *M*. *hominis* clearance was confirmed by PCR as described below. To generate dead, intact controls, *Tv* was counted, and then reconstituted in complete RPMI media, and rendered dead—but intact—by treatment at 65°C for 1 hour, followed by 3 freeze-thaw cycles [46]. Trichomonads were confirmed by microscopy to be immobile and intact and flow cytometry analysis using Zombie Red dead-cell exclusion dye (Biolegend) confirmed that trichomonads were not viable after this treatment.

### Primary human cell acquisition and culture

Primary human peripheral blood mononuclear cells (PBMC) were isolated from leukopacks or trima filters using Ficoll gradient. Blood was obtained from 32 de-identified, healthy donors from the UCLA Virology Core using a UCLA Institutional Review Board approved protocol. Monocytes were isolated based on adherence to plastic (for monocytes used in cytotoxicity experiments) or by Rosette Sep © negative isolation (Stem Cell technologies) (for monocytes used in cytokine secretion experiments). PBMC and monocytes were frozen directly after purification and used the day they were thawed. All experiments with PBMC and monocytes were done using RPMI 1640 media supplemented with 10% Fetal Bovine Serum, Pen/Strep, GlutaMAX, and MEM non-essential amino acids, (Life Technologies), and incubated at 37°C with 5% CO_2_.

### *In vitro* differentiation of human monocyte-derived macrophages (HMDM)

Primary human PBMC were isolated from a trima filters using Ficoll gradient using blood from donors at the UCLA Virology Core. Monocytes were separated based on adherence to plastic and were plated at 2.7 x 10^5^ cells/ml. 20 ng/ml of GM-CSF (Biolegend) was added for 4 days to induce macrophage differentiation. The differentiation was verified by staining with anti- CD14-PE (Invitrogen- MHCD14014) and an increase in forward scatter (size).

### Flow-Cytometry based cytotoxicity assay

Brooks et al. demonstrated that *Tv*- host cell co-cultures are suitable for flow cytometry analysis [47]. As the similar size of *Tv* and leukocytes confounds cell discernment via size and scatter properties alone, we utilized differential dye staining. PBMC were labeled with Carboxyfluorescein succinimidyl ester (CFSE) (Biolegend) at 1:2000 for 3 minutes according to the manufacturer’s instructions and then washed and plated at 2.5 x 10^5^ cells/well in 100 μl in u-bottom 96 well plates. *Tv* was labeled with Cell Tracker Blue © (Molecular Probes), according to the manufacturer’s instructions, and then allowed to recover in complete TYM media for 45 minutes- 2 hours. *Tv* was then reconstituted in complete RPMI media and added directly to wells containing PBMC at the indicated multiplicities of infection (MOI) for the indicated period of time. After incubation, cells were stained with anti-CD3 APC clone HIT3a and anti-CD19 PeCy7 clone HIN19 (both from Biolegend) at 1 μg/ml in FACS buffer (PBS with 5% FBS and 0.1% sodium azide) on ice for 30 minutes. Cells were then washed and resuspended in FACS buffer and analyzed within 2 hours on an LSR Fortessa © (Becton-Dickinson) at the UCLA Broad Stem Cell Research Center Flow Cytometry core facility. Directly before sample acquisition, 5 μl of Bright Count © counting beads (Life Technologies) was added to each sample. Data were analyzed using FlowJo (Treestar), and counts of each population (*Tv*, T-cell or B-cell) were determined according to the gating strategy is shown in S1 Fig. Cell counts were uniformly normalized to 2,000 beads, and percent death was calculated as ((# of B-cells in PBMC alone condition—# of B-cells in co-culture condition) / # of B-cells in PBMC alone condition) *100, for B-cells (same analysis was done for T-cells) or as ((# of parasites in parasites alone condition—# of parasites in co-culture condition) / # of parasites in parasites alone condition)*100 for *Tv*. Zombie Red dead-cell exclusion dye (Biolegend) was added to preliminary cytotoxicity experiments to assure that live cell gates based on forward and side scatter did not include dead cells (S2 Fig), and then found to be redundant since dead cells disappear from the live cell scatter plot completely. Zombie Red staining was therefore excluded in subsequent experiments for simplicity. For transwell cytotoxicity experiments, an HTS Transwell—96 well plate (Corning) with 0.4 μm polycarbonate membrane was used. PBMC were placed in the bottom, receiver plate, which was spun down and processed as described above after the co-culture. Two-tailed, unpaired student’s T-test was done to determine statistical significance between conditions, when relevant.

### Lactate dehydrogenase-based cytotoxicity assay

Monocytes isolated based on plastic adherence as described above were plated at 5 x 10^5^ cells/ well in 96-well flat-bottom plates. *Tv* was reconstituted in complete RPMI media as described above and added at the indicated MOI for the indicated period of time. After the co-culture, monocyte death was measured by determining mammalian-specific lactate dehydrogenase (LDH) release using the CytoTox-One © homogeneous membrane integrity assay (Promega) according to the manufacturer’s instructions. Samples were read on a Victor^3^ 1420 plate reader (Perkin-Elmer) to generate mean fluorescence intensity (MFI) values correlating with LDH presence in the supernatants. Percent death was calculated as (MFI from co-culture supernatants- MFI from live monocytes alone supernatants)/ (MFI from detergent solubilized monocytes alone supernatants–MFI from live monocytes alone supernatants) *100. Transwell cytotoxicity experiments were conducted as described above.

### Cytokine analysis

Human monocytes isolated by Rosette Sep © negative selection were plated at 5 x 10^4^ cells/ well in 96-well u-bottom plates. *Tv* was counted, and then reconstituted in complete RPMI media, and rendered dead, but intact as described above. Dead, intact *Tv* were then added to monocytes, or human monocyte derived macrophages (HMDM) at an MOI (multiplicity of infection) of 1, and allowed to incubate overnight (16 hours). Positive controls were 100 ng/ml LPS (sigma) 10 μg/ml poly (I:C) (Tocris) or 1000 U/ml IFN gamma (Biolegend). Subsequently plates were centrifuged and supernatants were harvested and frozen at -80°C. Supernatants were then thawed on ice and analyzed using Cytometric Bead Array (Becton-Dickenson) for IL-8, IL-6, IL-1β, TNFα, and IL-12 according to the manufacturer’s instructions. IL-6, IL-1β, and IL-12 were multiplexed, and IL-8 was measured separately on supernatants diluted 1:100. Data were analyzed using FlowJo (Treestar) to determine MFI, which was normalized to absolute concentrations according to a standard curve generated using lyophilized protein provided with the kit. IL-23 was measured using Legend Max Human IL-23 (p19/p40) ELISA kit with pre-coated wells (Biolegend) according to the manufacturer’s instructions. Wells were read using a Victor^3^ 1420 plate reader (Perkin-Elmer), and MFI was normalized as described above.

### *M*. *hominis* analysis in *T*. *vaginalis* strains

Dense 15ml cultures of *Tv* were lysed using a solution of 8M urea, 2% sarkosyl, 0.15M NaCl, 0.001M EDTA, and 0.1M Tris HCL pH7.5, and DNA was extracted using phenol: chloroform: ISA (Amresco), precipitated with isopropanol, and reconstituted in 10mM Tris pH8+ RNase. Then PCR was performed on *Tv* DNA using the following primer pairs: (1) *M*. *hominis* specific primers: 5’ CAA TGG CTA ATG CCG GAT ACG C 3’ and 5’ GGT ACC GTC AGT CTG CAA T 3’ [48] and (2) universal 16S rDNA primers: 5’ AGA GTT TGA TCC TGG CTC AG 3’ and 5’ GGA CTA CCA GGG TAT CTA AT 3’ (Greg James, PCR for Clinical Microbiology, 2010). PCR products were sequenced (Genewiz), and resultant sequences were aligned using NCBI nucleotide BLAST and ApE software and confidence peaks were examined in comparison to *M*. *hominis* sequence. These analysis were done on non-clonal *Tv* strains G3 and MSA1132 either cured of resident *M*. *hominis* strains or prior to curing with antibiotic treatment. Identical *M*. *hominis* sequences were derived using uncured strains in replicate experiments.

## Results

**Fig 1:**
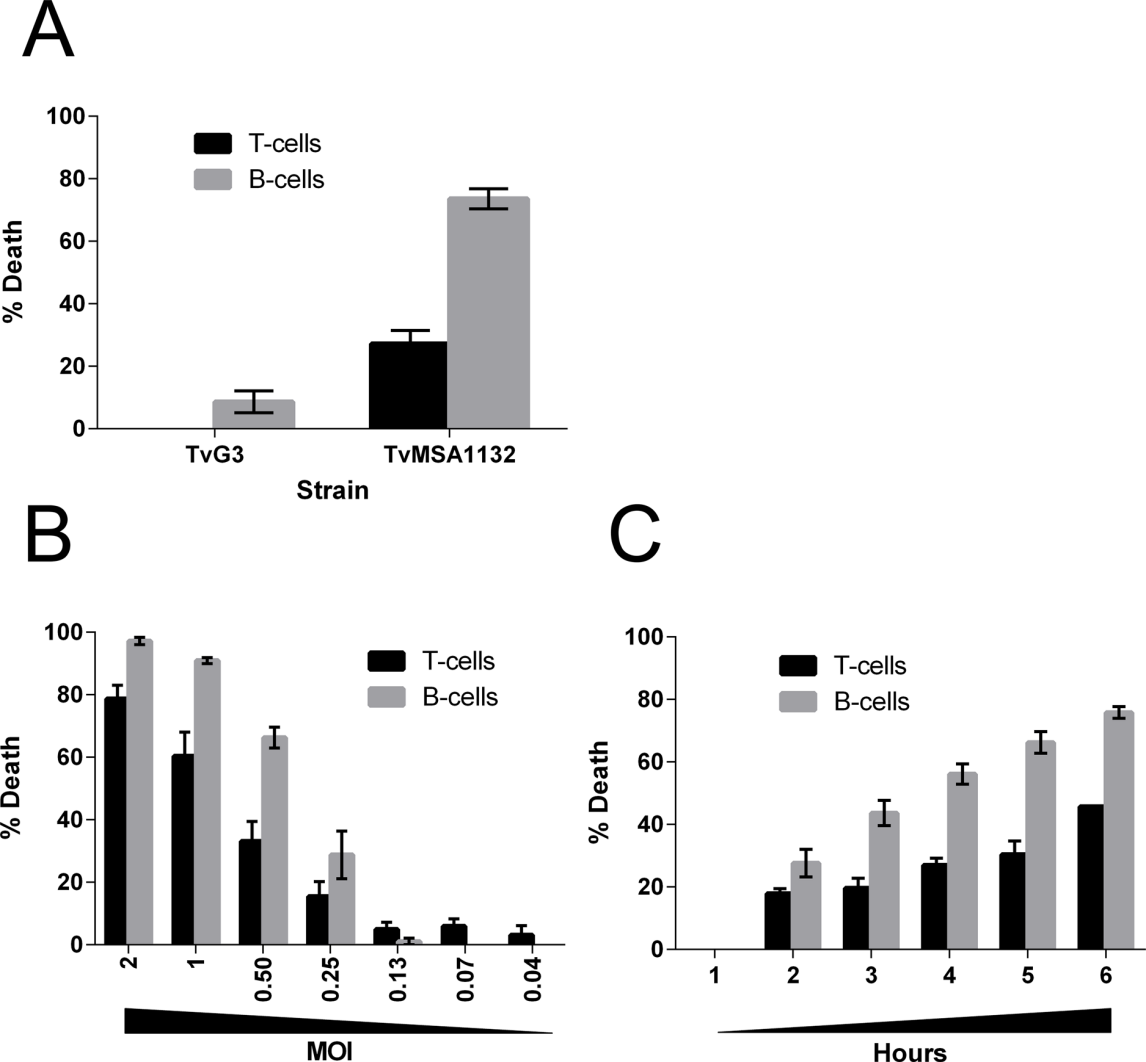
Lymphotoxic activity of *Tv*. **(A)** PBMC and *Tv* strains *Tv*G3 (laboratory adapted) or *Tv*MSA1132 (clinical isolate) were differentially labeled with CFSE or Cell Tracker Blue, respectively and co-cultured at an MOI of 0.5 for 4 hours. Cells were then stained with anti-CD3 and anti-CD19 to detect T-cells or B-cells, respectively, and lymphocyte death was assessed with flow cytometry analysis. **(B)** Killing of lymphocytes by *Tv*MSA1132 was assessed as in **(A)** at the indicated MOI for 4 hours. **(C)** Killing of lymphocytes by *Tv*MSA1132 was assessed as in **(A)** at an MOI 0.5 for the indicated period of time. All data in **(A-C)** are averages with standard deviation of triplicate wells and representative of at least 3 donors/ independent experiments.

*Trichomonas vaginalis* (*Tv*) is known to lyse cervical and prostate epithelial cells [2]. Since leukocytes are present in the genital mucosa [40], and in the lamina propria directly beneath the epithelial layer [41], we asked whether *Tv* is able to kill leukocytes. To assess this, we set up an *in vitro* co-culture system of *Tv* with primary human peripheral blood mononuclear cells (PBMC), and then determined numbers of surviving cells using flow cytometry following the co-culture. Prior to the co-culture, *Tv* were labeled with Cell Tracker Blue © (CTB), and PBMC were labeled with carboxyfluorescein succinimidyl ester (CFSE). After co-culture, wells were stained with anti-CD3 and anti-CD19 to identify T-cells and B-cells, respectively. Directly before flow cytometry analysis, counting beads were added to wells for sample-to-sample volume normalization. Counts of live CTB+ CFSE- (*Tv*), CFSE+CD3+ (T-cells) and CFSE+CD19+ (B-cells) events were then determined and normalized to PBMC alone, or *Tv* alone controls to calculate percent death of each population. Using this system, we first tested a common laboratory adapted strain of *Tv* (G3) [49] compared to a relatively recent clinical isolate of *Tv* (MSA1132) [2]. We observed that clinical strain MSA1132 was able to kill ~30% of T cells and ~70% of B-cells in the co-culture, using a multiplicity of infection (MOI) of 0.5 (1:2 *Tv*: host cell ratio). On the other hand, *Tv* lab strain G3 did not kill T-cells under these conditions and demonstrated only minimal killing of B-cells (Fig 1A).

Having determined that *Tv*MSA1132 is cytotoxic towards lymphocytes, we next addressed the efficiency and kinetics of *Tv* lymphotoxic activity. The amount of *Tv* needed to lyse lymphocytes was determined by performing the cytotoxicity assay at various MOI. We found moderate levels of lymphocyte death occurring at MOI of 0.25 (1:4, *Tv*: host cells), whereas at MOI 2 (2:1, *Tv*: host cell), we found almost complete killing of B-cells and ~75% killing of T-cells (Fig 1B). Lymphocyte killing requires live parasites, as co-cultures containing dead, intact *Tv* at the same MOI did not result in any lymphocyte death (S3 Fig). Next, we determined how fast *Tv* killing of lymphocytes occurs by performing cytotoxicity assays at an intermediate MOI (0.5), and assessing lymphocyte death at various time points during the co-culture. We found that lymphocyte death was not rapid, and required 2 hours to achieve low levels, and 5–6 hours to achieve relatively high levels of death (Fig 1C). Using *Tv*MSA1132 as a model clinical strain, these data indicate that some clinical strains of *Tv* possess lymphotoxic activity and require several hours and high parasite titers to achieve maximal killing; nevertheless moderate lymphotoxic effects are observed at shorter time points and lower parasite titers. Also, while there was some donor-to-donor variability in susceptibility to *Tv* cytotoxicity, B-cells were significantly more susceptible (S4A Fig).

**Fig 2:**
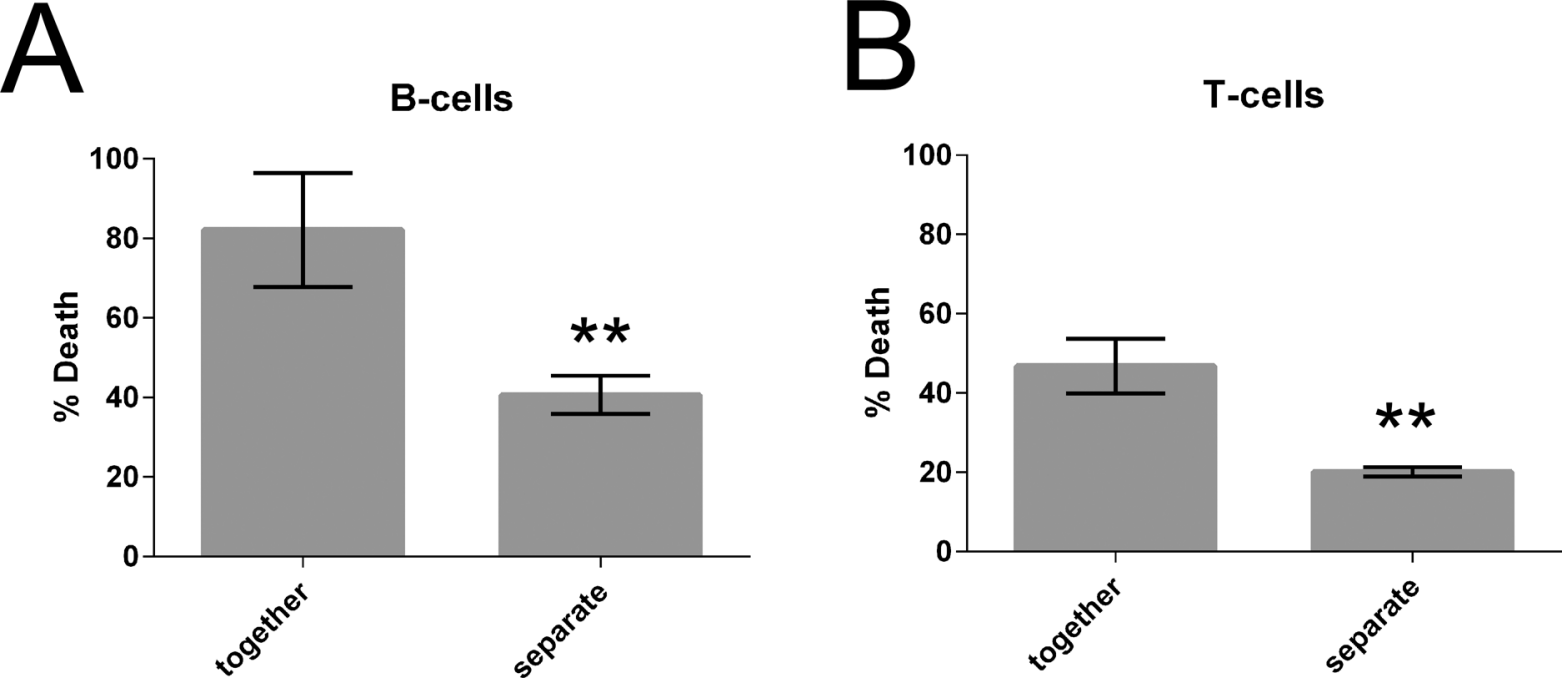
*Tv* lymphotoxic activity is mediated by contact dependent and soluble factors. PBMC and *Tv*MSA1132 were co-cultured and lymphocyte death was assessed as described in Fig 1 at an MOI of 0.5 for 4 hours in a transwell apparatus. PBMC were placed in the bottom chamber and *Tv*MSA1132 were placed either with PBMC in the bottom chamber (together) or in a top chamber with shared media, but separated by a 0.4 μm porous membrane (separate). Percent death in the bottom chamber was then assessed for B-cells **(A)** and T-cells **(B)**. Data shown are averages of triplicate wells with standard deviation, and are representative of 4 donors and 2 independent experiments.

*Tv* likely comes into direct contact with lymphocytes at the luminal side of the vaginal mucosa and also in the lamina propria after tissue invasion associated with lysis of the epithelial layer. However, *Tv* killing of lymphocytes could have an extended effect in tissues if it were mediated by soluble factors. We therefore asked whether *Tv* lymphotoxic activity was contact-dependent, or mediated by soluble factors by performing our cytotoxicity assays utilizing a trans-well insert system with a 0.4 μm porous membrane. As *Tv* is 7–10 μm in diameter, the parasite cannot pass through [2]. In conditions where PBMC and *Tv* were cultured together, death of T-cells and B-cells was observed at levels similar to that shown in Fig 1. However, when *Tv* and PBMC were placed in separate chambers there was an approximately 2-fold decrease in death of T-cells and B-cells (Fig 2A and 2B). The significant difference in lymphotoxic activity observed when parasite and lymphocytes were in the same chamber or separated by a membrane indicates that killing of B-cells and T-cells by *Tv* is mediated by both contact dependent and soluble factors.

**Fig 3:**
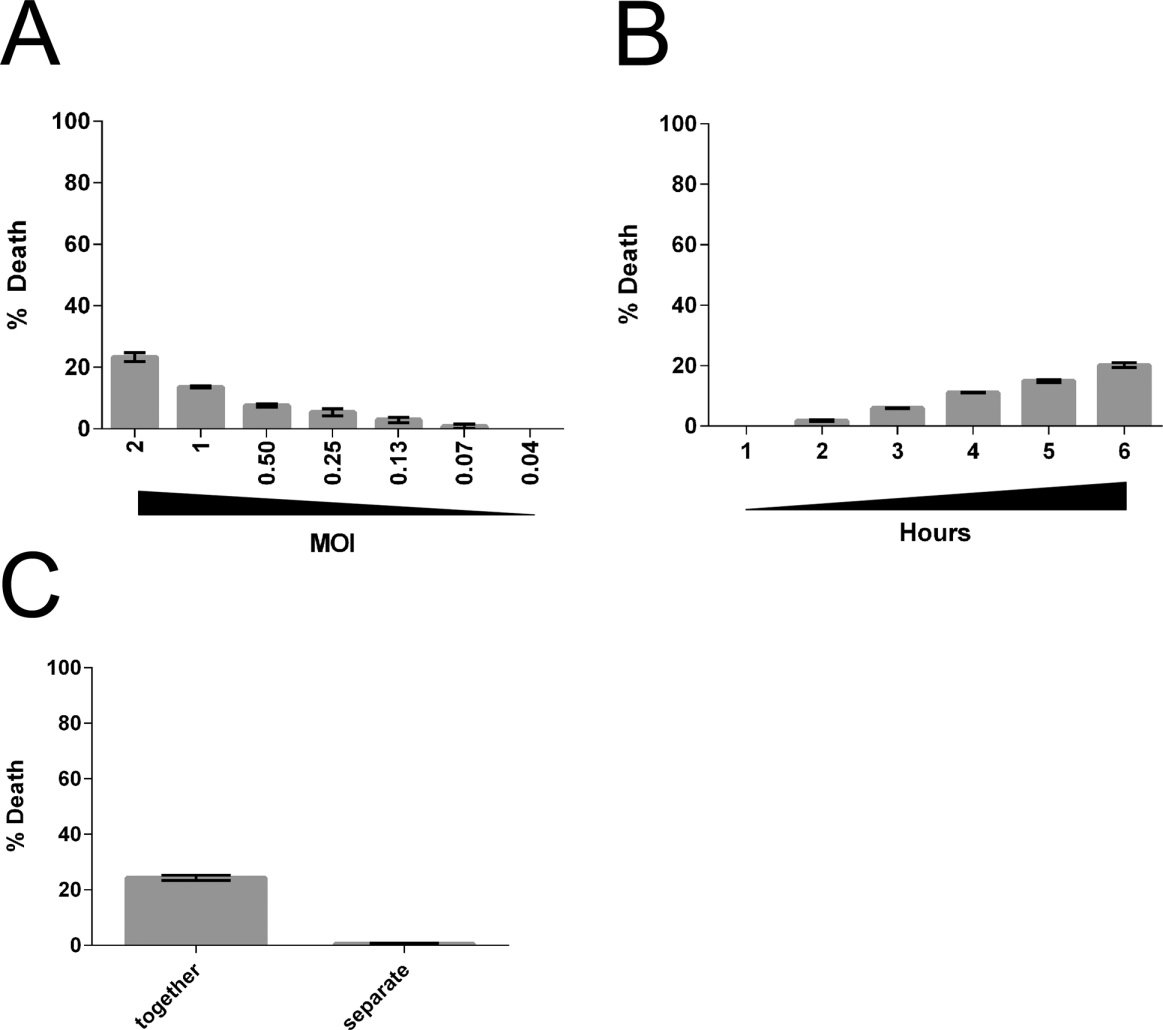
*Tv* cytotoxicity towards monocytes is inefficient and contact-dependent. **(A)** Primary human monocytes were co-cultured with *Tv*MSA1132 at the indicated MOI. Monocyte death was assessed by detecting the release of mammalian lactate dehydrogenase (LDH) into culture supernatants after 4 hours. **(B)** Primary human monocytes were co-cultured with *Tv*MSA1132 at MOI 0.5 for the indicated times and percent death was determined. **(C)** Killing of monocytes by *Tv*MSA1132 at an MOI of 2 was determined using a transwell apparatus as described in Figure legend 2, except monocytes were used instead of lymphocytes. LDH was measured in culture supernatants after 4 hours. All data in shown **(A-C)** are averages of triplicate wells with standard deviation, and are representative of at least 3 donors/independent experiments.

We next asked whether *Tv* can lyse monocytes. To avoid variability arising from adherence of activated monocytes to plastic, we measured levels of lactate dehydrogenase (LDH) released in the culture supernatants upon lysis of monocytes, instead of using flow cytometry. *Tv* killing of monocytes was found to be inefficient, with only ~20% death of monocytes observed at an MOI of 2 (2 *Tv*:1 monocyte) (Fig 3A). A 1–6 hour time course was used to assess the kinetics of killing, and death was found to be linear over 6 hours (Fig 3B). It is notable that prolonged co-incubation did not significantly increase monocyte death even after 24 hour. While there was some donor-to-donor variability, *Tv* killing of monocytes was always less efficient than that of lymphocytes (S4B Fig). To determine whether *Tv* lysis of monocytes is contact-dependent, cytotoxicity assays were performed using the transwell system. We found that unlike that observed for killing of lymphocytes (Fig 2), separation of *Tv* from monocytes almost completely abolished cytotoxic activity (Fig 3C). Together these data indicate that *Tv* killing of monocytes is inefficient and primarily contact-dependent.

**Fig 4:**
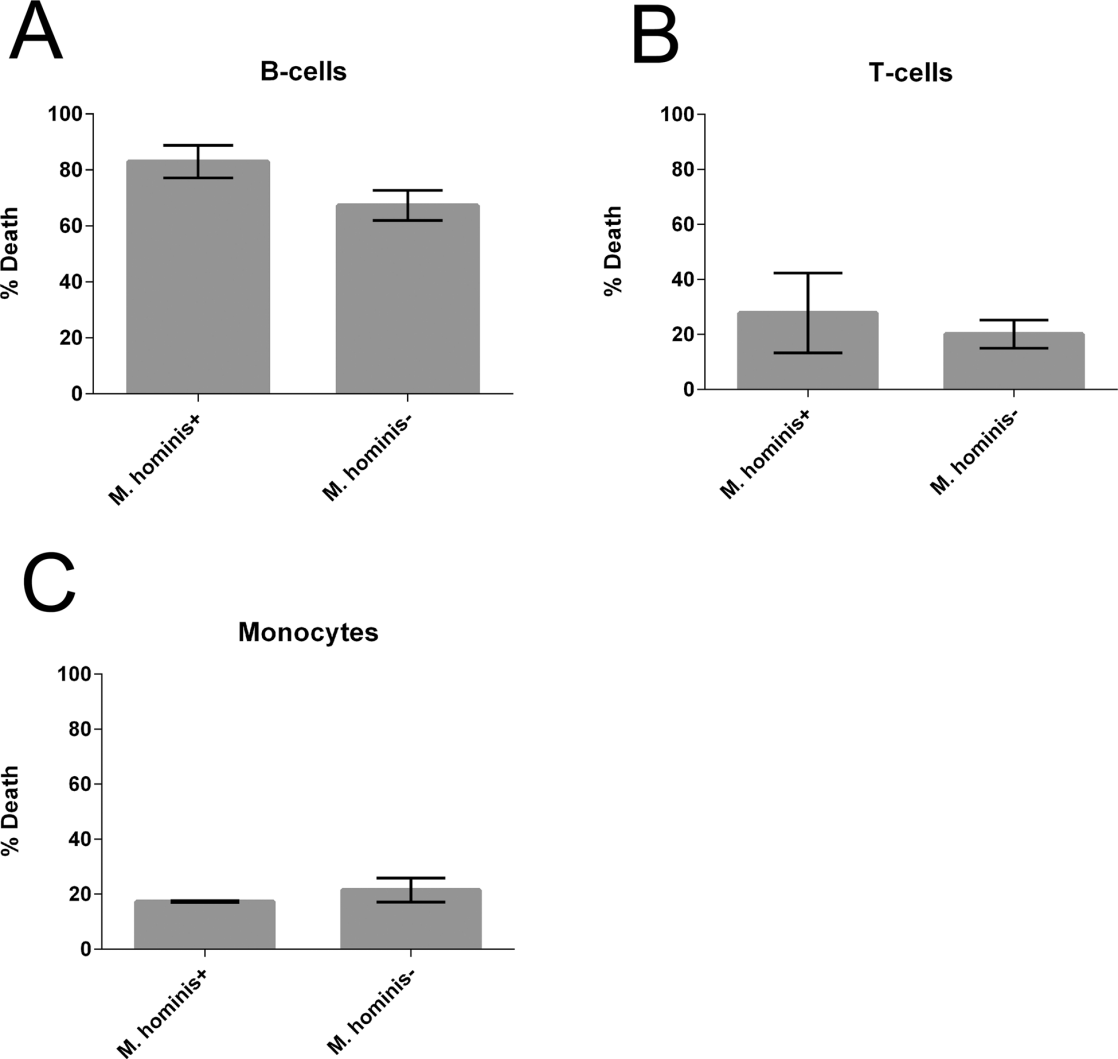
*Tv* symbiont *Mycoplasma hominis* does not affect *Tv* leukotoxic activity. Killing of B-cells **(A)**, T-cells **(B)**, or monocytes **(C)** by *TvMSA*1132 was determined at an MOI 0.5 for 4 hours. Prior to the cytotoxicity assay, *Tv*MSA1132 was either cultured in the presence (*M*. *hominis -*) or absence (*M*. *hominis +*) of additional antibiotics. Data shown are average of triplicate wells with standard deviation, and are representative of 3 donors/ independent experiments.

Since symbionts often increase fitness of their hosts, we next asked whether a common symbiont of Tv, *M*. *hominis*, affects the ability of *Tv* to kill leukocytes. *Tv*MSA1132 naturally contains *M*. *hominis*, thus to generate isogenic strains that either harbor the symbiont or not, we cultured *Tv*MSA1132 for 1 week either untreated, or in the presence of chloramphenicol and tetracycline. PCR using *M*. *hominis*-specific primers was then conducted to confirm that *M*. *hominis* was undetectable in the culture treated with additional antibiotics (S5 Fig). Furthermore, sequencing the products generated by PCR using universal 16S bacterial primers on our untreated cultures confirmed that *M*. *hominis* is the only bacterial species present in the untreated culture. We then used the untreated (*M*. *hominis*+) and antibiotic treated (*M*. *hominis*-) strains side-by-side in leukocyte cytotoxicity assays. We found that the ability to kill T-cells, B-cells or monocytes was not significantly different, indicating that *M*. *hominis* does not confer greater leukotoxic activity to *Tv* (Fig 4A–4C).

**Fig 5:**
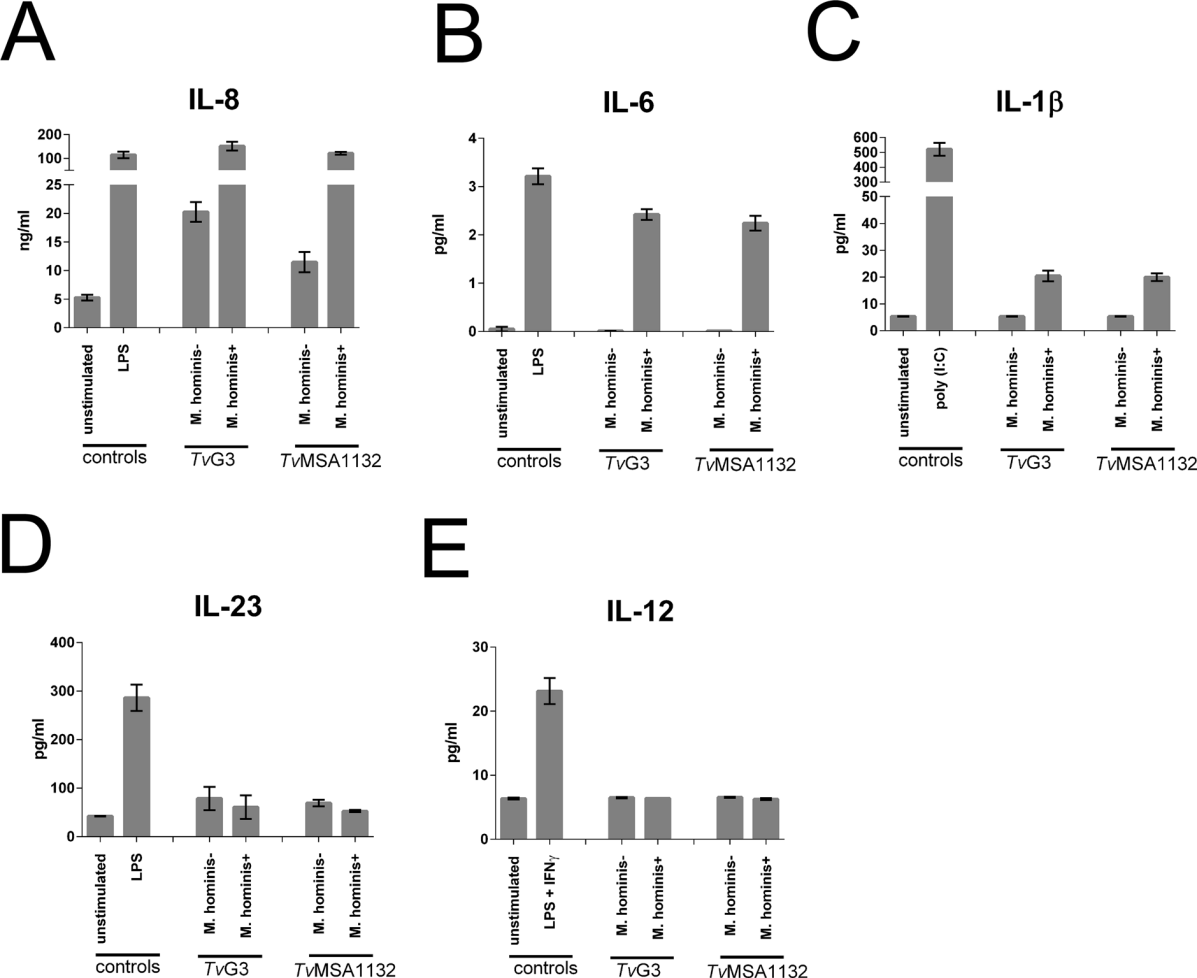
Induction of cytokine secretion from human monocytes by *Tv* is largely dependent on the presence of *Mycoplasma hominis* and is dominated by IL-8 secretion. Primary human monocytes were cultured with either dead intact *Tv*G3, *Tv*MSA1132, unstimulated, or treated with LPS (**A, B, D**) or poly (I:C) **(C)** or LPS and IFNγ **(E)** for 16 hours. Prior to killing, *Tv* strains were either untreated (*M*. *hominis*+) or treated with additional antibiotic to clear the symbiont (*M*. *hominis*-). Supernatants were collected and the indicated cytokines were measured using Cytometric Bead Array (CBA) or ELISA. Data shown are average of triplicate wells with standard deviation, and are representative of 3 donors/ independent experiments.

Having observed that *Tv* is potentially able to modulate immune responses by killing lymphocytes, we next asked the converse question: what type of immune response is mounted against *Tv*? To address this, we assayed for the presence of several cytokines secreted from primary human monocytes after overnight exposure to *Tv*. To prevent *Tv* killing of monocytes from affecting the results, we made dead, intact preparations of *Tv* before co-culture and confirmed that no monocyte death occurred after incubation with dead *Tv*. Fiori and colleagues recently showed that *M*. *hominis* dramatically increased the amount of pro-inflammatory cytokine induced from a human macrophage-like cell line [38]. We were able to reproduce these results using primary human monocyte derived macrophages (HMDM) (S6 Fig). We therefore compared cytokine responses induced from untreated (*M*. *hominis*+) or additional antibiotic treated (*M*. *hominis*-) strains using fresh, naïve human monocytes. We also compared the responses against the laboratory adapted (*Tv*G3) versus the clinical (*Tv*MSA1132) strain. Levels of cytokines known to support Th1 responses (IL-12) and Th17 responses (IL-6, IL-1β, and IL-23) were assessed. IL-8, a broadly inflammatory, neutrophil-recruiting chemokine previously reported to be highly secreted following *Tv* encounter [31,32,37] was also measured. All strains of *Tv* tested induced IL-8 secretion over background (Fig 5A). Notably, the presence of *M*. *hominis* greatly increased IL-8 induction, relative to isogenic strains lacking *M*. *hominis* (Fig 5A), and enabled induction of IL-6 and IL-1β which were otherwise not detectable in response to *Tv* alone (Fig 5B and 5C). In contrast, neither *M*. *hominis* positive nor negative parasites induced detectable IL-23 or IL-12 secretion (Fig 5D and 5E). No difference was observed in the immunogenicity of laboratory-adapted strain *Tv*G3 compared to clinical strain *Tv*MSA1132 (Fig 5A–5E). Together these analyses indicate that IL-8 is the dominant cytokine response to *Tv*, both in the presence and absence of *M*. *hominis*. The data also show that the symbiont *M*. *hominis* greatly increases the induction of inflammatory cytokines IL-6 and IL-1β, in addition to IL-8. This increased immunogenicity may be instrumental in triggering adaptive immune responses that would not normally be mounted against *Tv* in the absence of the symbiont.

## Discussion

Despite the numerous inflammatory complications associated with *Tv* infection, how *Tv* interacts with the cells of the host immune system is not well characterized. Furthermore, partner re-infection after treatment for trichomoniasis indicates a lack of effective adaptive immunity to *Tv*. Using primary human leukocytes, we have demonstrated that *Tv* has leukotoxic activity, that IL-8 secretion dominates the primary cytokine response to *Tv* infection, and that the *M*. *hominis* symbiont is likely to play a major role in shaping more robust and diverse inflammatory responses to *Tv*. These results form a foundation for the dissection of interactions between *Tv* and the cells of the human immune system. These studies are the first to examine how primary human leukocytes respond to *Tv*, and to assess *Tv* leukotoxic activity, with attention to strain specificity, host cell-preference, timing, and dosage. These analyses have also interrogated the contribution of the symbiont *M*. *hominis* to the pathogenesis and immunogenicity of *Tv* using primary immune cells. We found that immune responses against *Tv* may be modulated by leukotoxic activity of the parasite as well as the presence of *M*. *hominis*. These results suggest potential explanations for the considerable variability in *Tv* clinical presentation, pathogenicity, and inflammatory sequelae.

The leukotoxic activity of *Tv* reported here may be important in subverting immune responses, or in modulating the leukocyte repertoire in the vaginal mucosa, where leukocytes may control concomitant STIs and commensal micro-organisms [41]. We sought to determine which cells among PBMC are primary targets of the parasite and found that *Tv* demonstrates a preference for killing B-cells, followed by T-cells, and is very inefficient at killing monocytes. Interestingly, while the cell-type preference was maintained in all donors tested, there was some variability in overall susceptibility of leukocytes to *Tv*- mediated killing (S4 Fig), which could account for variation in symptoms and sequelae in the clinic. It is interesting that B-cells are the most vulnerable leukocyte in the presence of *Tv*, as humoral immunity is likely to be important in host defense against *Tv*: a large extracellular eukaryotic pathogen. Antibodies against *Tv* can be detected in sera and vaginal washes of infected individuals [1,34,50], indicating that humoral immunity is formed against the parasite. Furthermore, *Tv* strains that do not harbor the symbiont *M*. *hominis* do not induce detectable levels of IL-1β, IL-6 or IL-12 secretion from monocytes (Fig 5B, 5C and 5E), suggesting formation of default Th2 responses to *Tv*, at least in the absence of symbiont *M*. *hominis*. Since ~50% *Tv* clinical isolates lack *M*. *hominis* [27,30], Th2 responses could predominate in these cases. Killing of antibody-producing B-cells (such as those at the mucosa secreting IgA [51] could therefore be a way for the parasite to subvert immune clearance. Interestingly, *Tv* proteases are reported to cleave IgG and secretory IgA [52] and Tv exhibits antigenic variation [53], both consistent with a model of humoral immunity subversion as an evolved behavior of *Tv* to survive in its host. We also found that approximately 50% of *Tv* leukotoxic activity against B-cells was mediated by soluble factors, indicating that the parasite may kill B-cells even if it does not come into direct contact with them, potentially allowing for a broader effect of this anti-B cell activity. Contact dependent killing was also observed, consistent with previous work showing that human PBMC can be phagocytosed by *Tv* [54].

Moderate cytotoxic activity of *Tv* against T-cells was also detected, albeit lower than that exhibited towards B-cells, again mediated by both contact-dependent and soluble factors (Figs 1 and 2). *Tv*-antigen-induced proliferation of PBMC from infected women [36] indicates that T-cell responses are formed against *Tv in vivo*. *Tv* killing of T-cells could therefore potentially subvert anti-*Tv* immune responses as well as affect the T-cell repertoire in the such that control of concomitant STIs or commensals is dysregulated. Indeed, Tv infection is associated with dysbiosis of microbiota in the FRT and bacterial vaginosis [55–57]. Since T-cell polarization is a delicate and multi-factorial process involving both positive and negative feedback, *Tv* killing of T-cells could have more complex downstream implications in anti-*Tv* immunity, on other vaginal microflora, and on mucosal inflammation.

We observed both contact-dependent and contact-independent leukotoxic activity for the clinical isolate *Tv*MSA1132. Previously demonstrated phagocytosis of leukocytes [42] could account for or contribute to the contact-dependent leukotoxic activity observed. Alternatively, the cytotoxicity could be mediated almost entirely by soluble factors, but require close proximity; concentrations of pH and secreted effectors being effectively higher in such a microenvironment. Contact-independent killing of leukocytes by *Tv* has not been previously described. However soluble, extracellular *Tv* cysteine proteases have been isolated [58,59] and several studies implicate them in *Tv* cytolytic mechanisms [60–63]. In addition, several genes with homology to known pore-forming toxins are present in the *Tv* genome [49]. Future work to determine the identity of soluble factors involved in *Tv* contact-independent killing of lymphocytes will be important to understand the mechanism underlying this mode of host cell cytotoxicity.

The observation that the recent clinical isolate *Tv*MSA1132 demonstrated dramatically enhanced lymphotoxic activity compared to the common laboratory adapted strain *Tv*G3 (Fig 1A) is notable. We have previously shown that *Tv* lysis of prostate and vaginal epithelial cells, which is strictly contact-dependent, is highly variable among different *Tv* strains [2]. Strain differences in contact-independent modes of lysis, as demonstrated here, reveal an added layer of strain variation in pathogenic behavior, underscoring the value of using clinical strains for studies of *Tv* molecular pathogenesis, and highlighting a likely reason for the considerable clinical variability in trichomoniasis presentation and outcomes. Unfortunately, the clinical symptoms of the patient from which *Tv*MSA1132 was isolated are not available to allow direct comparison of host cell toxicity *in vitro* with clinical outcomes. Variability between *Tv* and *M*. *hominis* strains and their host cell-specific interactions may also explain why in this study we did not find differences between *M*. *hominis* positive and negative *Tv* cultures in their ability to kill leukocytes (Fig 4), in contrast to the enhancement of epithelial cell lysis conferred to *Tv* by *M*. *hominis* observed by Vancini and colleagues [64]. This further supports a model of diverse mechanisms underlying *Tv* cytotoxicity that may be strain and host cell type dependent.

In contrast to the efficient killing of lymphocytes by *Tv*MSA1132, monocytes were refractory to killing and the cytotoxicity that was observed was almost exclusively contact-dependent (Fig 3). Variation in the ability of *Tv* to kill host cells may be dependent on specific host-cell factors that are enriched on epithelial cells [2] and B-cells, and are present at lower levels on T-cells and monocytes. Only one host-cell receptor for mediating *Tv* interaction with host cells, galectin-1, has been identified. However, knock-down of galectin-1 expression in epithelial cells abrogated only ~20% of *Tv* adherence, indicating that adherence and contact-dependent cytolysis is a multi-factorial process [44]. Future studies aimed at identifying novel host molecules that confer vulnerability to contact-independent *Tv* killing will be important to understanding mechanisms of *Tv* leukotoxic activity.

A lack of robust cytotoxic activity towards monocytes (Fig 3) indicates that *Tv* does not subvert cytokine secretion from myeloid cells by killing the producers. Rather, cytokine secretion by monocytes is likely to proceed even in the presence of live, active parasites. In agreement with Fiori and colleagues [38] we found cytokine secretion by monocytes to be remarkably affected by the presence of the symbiont *M*. *hominis* (Fig 5). The absence of inflammatory cytokine production stimulated from monocytes in the presence of *M*. *hominis*-free *Tv* strains suggests that Th2 responses may be formed in cases where *Tv* strains are *M*. *hominis* negative. As *Tv* is an extracellular pathogen, and no IL-12 was induced in response to any *Tv* strain tested, it seems unlikely that Th1 responses are formed against the parasite. However, in the presence of *M*. *hominis* or other bacterial or viral antigens to provide co-stimulation in the milieu *in vivo*, Th17 responses specific to *Tv* may form. In humans, IL-1β, IL-6, and IL-23 support the formation or persistence of Th17 cells [65]. We saw that IL-1β and IL-6 were stimulated by *M*. *hominis*-infected *Tv*. In contrast to that observed by Fiori et al., we did not detect significantly high amounts of IL-23 in response to *M*. *hominis* + *Tv*, which potentially highlights a difference between the THP-1 macrophage cell line that Fiori et al. used and our primary human monocyte system, or that Fiori et al. used live trichomonads as opposed to our use of dead-intact trichomonads as stimulant. Regardless, IL-23 could be present in *in vivo* milieus as a result of other commensals or concomitant STIs. Indeed, IL-17 has been shown to be a major player in the immune response against the related parasite *Giardia lamblia* [66–68], and IL-22, a common cytokine associated with Th17 responses has been detected in vaginal secretions from *Tv* infected patients [69]. Th17 responses are common in the mucosa, where they respond to extracellular pathogens by recruiting neutrophils and repairing damaged epithelia [70,71], consistent with FRT neutrophilia and epithelial damage associated with *Tv* infection.

The commensal lactobacilli, dominant in ~75% of women’s vaginal microbiomes, have been shown to modulate *Tv* pathogenic properties *in vitro* [72]; it is conceivable that moieties present on commensal bacteria sharing a niche with *Tv* could additionally affect cytokine responses during *Tv* infection. Recently, additional *Mycoplasma* species associated with clinical strains of *Tv* have been discovered [73]. Known symbionts, commensals, and concomitant STIs are likely only the “tip-of-the-iceberg;” many other yet undefined organisms may contribute to the complex ecosystem with which *Tv* co-operates and contends. Meta-analysis of the FRT microbiome in the context of *Tv* infection, as well as development of suitable *in vivo* models will be instrumental to more fully appreciate this diversity and test hypotheses about *Tv* clearance and inflammation with a more holistic approach.

We found that both *Tv* strains examined, regardless of *M*. *hominis* status, induce IL-8 secretion (Fig 5) [1,31,32,37]. IL-8 is a pleiotropic cytokine [74], with a main function of recruiting neutrophils, consistent with that observed in trichomoniasis in the clinic. Furthermore, work in mouse models has shown that prevention of neutrophil influx is needed to establish infection [43], and primary human neutrophils were shown to swarm and attack *Tv in vitro* [32] suggesting that neutrophils are crucial for control of *Tv* infection. However, the molecular determinants of neutrophil-*Tv* interactions are not characterized. Neutrophils have a range of highly inflammatory and destructive behaviors [75], and can even contribute to cancer microenvironments [74]. It is possible that neutrophils may contribute to the associations of reproductive complications, inflammatory pathologies, and cancers of the reproductive tract with *Tv*.

These studies shed light on potential reasons for variability in *Tv* clinical presentation and associated complications, suggest potential immune subversion strategies of the parasite, and may help to inform future immunotherapy interventions. A better understanding of how *Tv* interacts with the immune system will also potentiate the design of immunotherapies to aid in scenarios of antibiotic resistance or to mitigate damaging inflammatory processes induced by *Tv* infections.

## Supporting Information

S1 Fig Gating strategy used for flow cytometry- based cytotoxicity assays. (Top panels) Total wells were analyzed for forward scatter vs. side scatter and beads, and live cells were gated on. Beads were further gated based on A-405 positivity to more accurately ensure their identity. (Middle panels) Live cells were further sub-gated based on CFSE+ to gate on leukocytes only (*Tv* excluded). (Bottom panels) Leukocytes were then further gated based on CD19 and CD3 positivity to identity B-cells and T-cells, respectively.
(TIF)

S2 Fig Viability analysis of leukocytes after *Tv* co-culture. Total PBMC from live cell gates (shown in S1 Fig) were analysed for Zombie Red expression to rule out that significant T-cells or B-cells occurring in live cell gates had compromised membranes.
(TIF)

S3 Fig Dead trichomonads do not cause death of PBMC. To assure that PBMC death observed after co-cultures with *Tv* was specific to live Tv-mediated mechanisms, we co-cultured PBMC with dead, intact *Tv* as a control. We did not observe any decrease in counts of viable PBMC after co-culture with dead, intact *Tv*. Data shown are from *Tv* co-culture with PBMC at MOI 0.5 for 4 hours and representative of multiple experiments.
(TIF)

S4 Fig Donor variation in susceptibility to *Tv*- mediated killing. % death is shown for all donors used in the study, at MOI 0.5 *Tv*MSA1132 for 4 hours. B-cell versus T-cell susceptibility was compared using paired (donor-matched), student’s T-test.
(TIF)

S5 Fig *Mycoplasma hominis* is undetectable in *Tv*MSA1132 treated with antibiotics and is the only bacteria present in untreated *Tv*MSA1132 cultures. Chloramphenicol/ tetracycline treated (*Tv*MSA1132 Cm/Tet) parasites were analyzed for the presence of bacterial symbionts using universal 16S primers designed to amplify a conserved region of bacterial 16S rDNA. The designed primers amplify a fragment of 834 bp. (Cm: chloramphenicol; Tet: tetracycline) specifically in *Tv*MSA1132 untreated parasites. DNA sequencing of the uncloned, amplified 16S bacterial rDNA fragment from untreated *Tv*MSA1132 was analyzed by BLAST analyses of Genbank. Only one sequence, with 100% homology to 16S region of *M*. *hominis* was detected. The sequence matched the following accession numbers with 100% identity: (all strains of *M*. *hominis*) CP009652.1, JN935871.1, NR113679.1, NR041881.1, FP236530.1, AF443616.3, AF443617.3, AJ002268.1, AJ002267.1, AJ002266.1, and AJ002265.1.
(TIF)

S6 Fig *M*. *hominis*+, but not *M*. *hominis*- strains stimulate cytokine release from human monocyte- derived macrophages. **(A)** Differentiation of human monocytes to macrophages (HMDM) was verified by the expression of CD14 and the increase in size. **(B)** HMDM were either unstimulated, treated with LPS, or cultured with heat-inactivated *Tv*G3 for 16 hours. Supernatants were collected and the indicated cytokines were measured using CBA. Data shown are average of triplicate wells with standard deviation, and are representative of 3 donors/ independent experiments.
(TIF)
