# Supplementary figures and images for: Leukocyte Lysis and Cytokine Induction by the Human Sexually Transmitted Parasite Trichomonas vaginalis
Source: PLoS Negl Trop Dis. 2016 Aug 16;10(8):e0004913. doi: 10.1371/journal.pntd.0004913 (PMC4986988; doi:10.1371/journal.pntd.0004913)

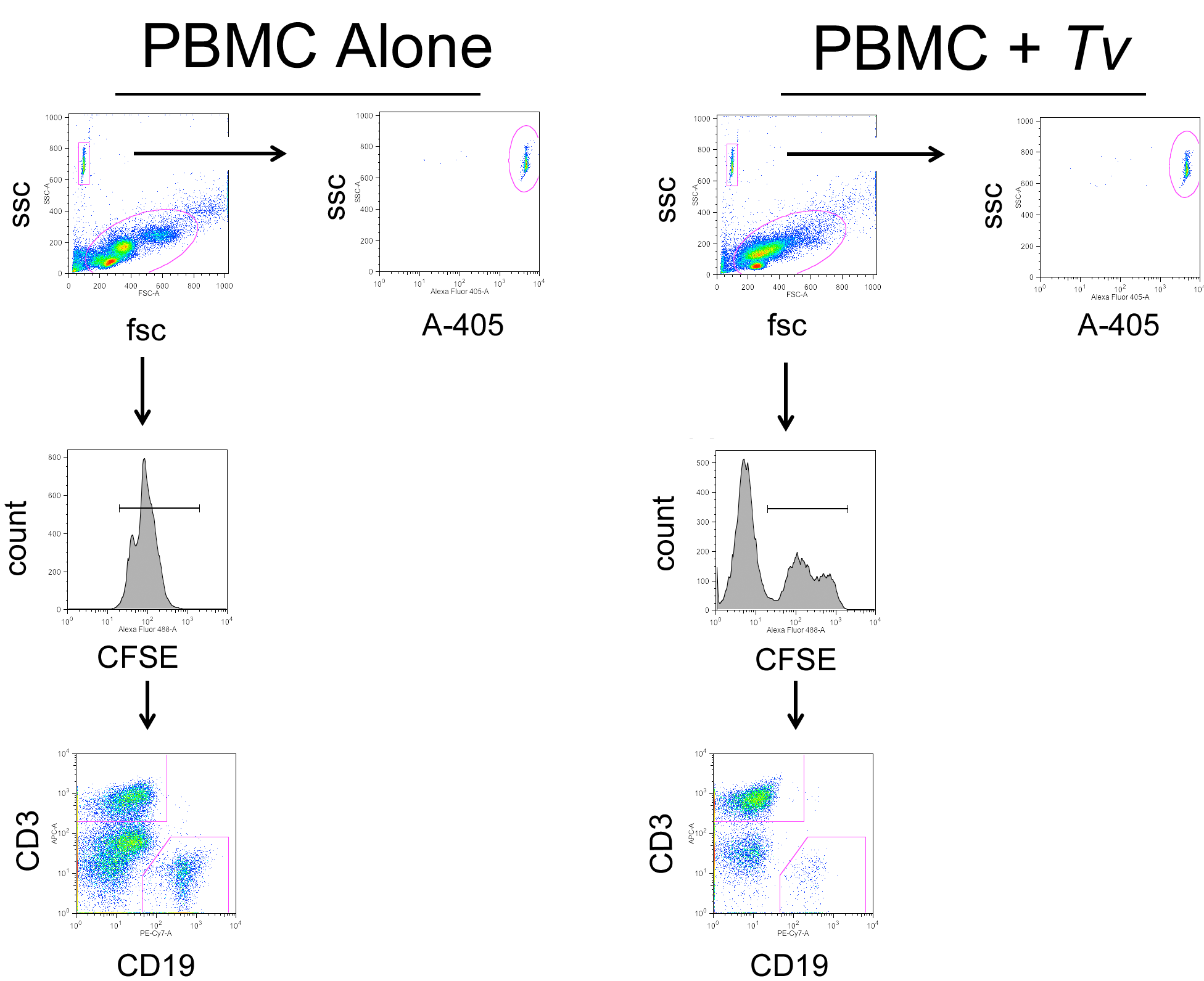

Supplement: S1 Fig — (Top panels) Total wells were analyzed for forward scatter vs. side scatter and beads, and live cells were gated on. Beads were further gated based on A-405 positivity to more accurately ensure their identity. (Middle panels) Live cells were further sub-gated based on CFSE+ to gate on leukocytes only (Tv excluded). (Bottom panels) Leukocytes were then further gated based on CD19 and CD3 positivity to identity B-cells and T-cells, respectively. (TIF) [file pntd.0004913.s001.tif]

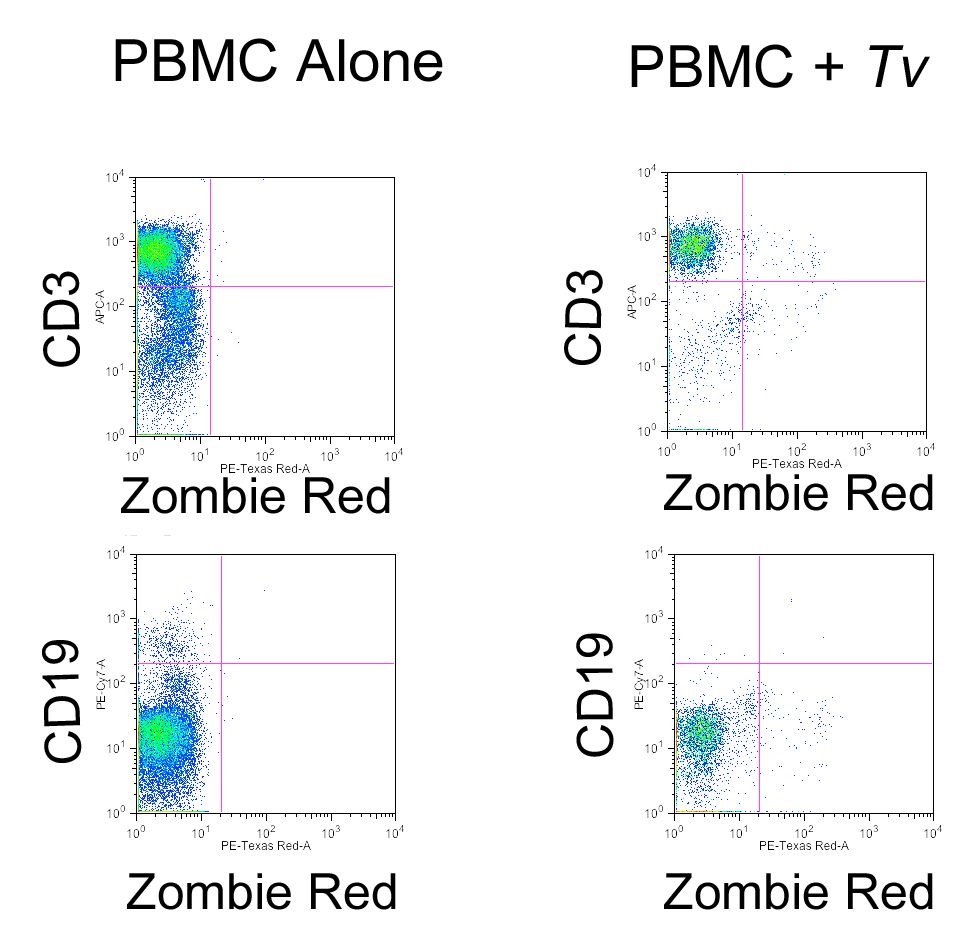

Supplement: S2 Fig — Total PBMC from live cell gates (shown in S1 Fig) were analysed for Zombie Red expression to rule out that significant T-cells or B-cells occurring in live cell gates had compromised membranes. (TIF) [file pntd.0004913.s002.tif]

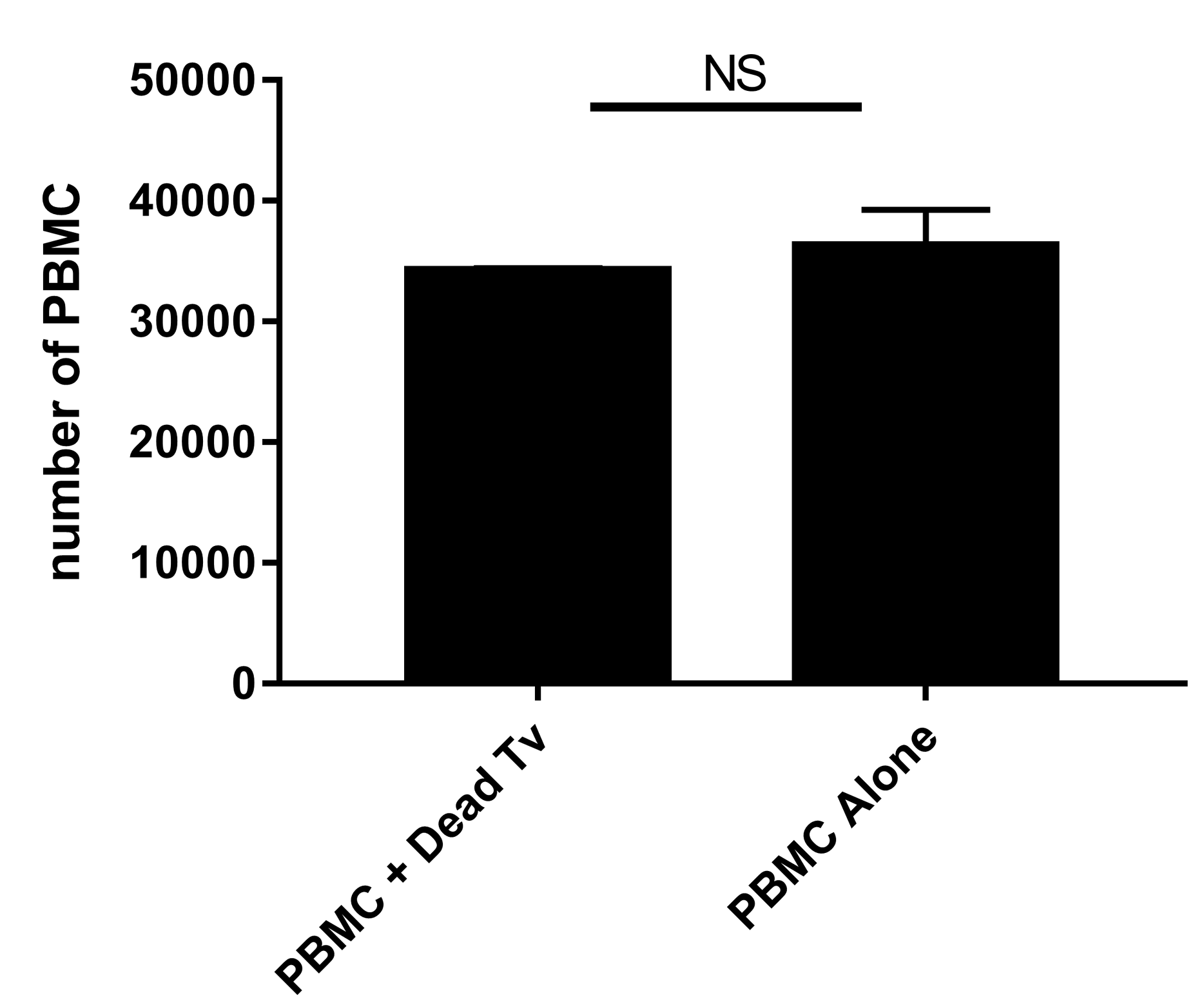

Supplement: S3 Fig — To assure that PBMC death observed after co-cultures with Tv was specific to live Tv-mediated mechanisms, we co-cultured PBMC with dead, intact Tv as a control. We did not observe any decrease in counts of viable PBMC after co-culture with dead, intact Tv. Data shown are from Tv co-culture with PBMC at MOI 0.5 for 4 hours and representative of multiple experiments. (TIF) [file pntd.0004913.s003.tif]

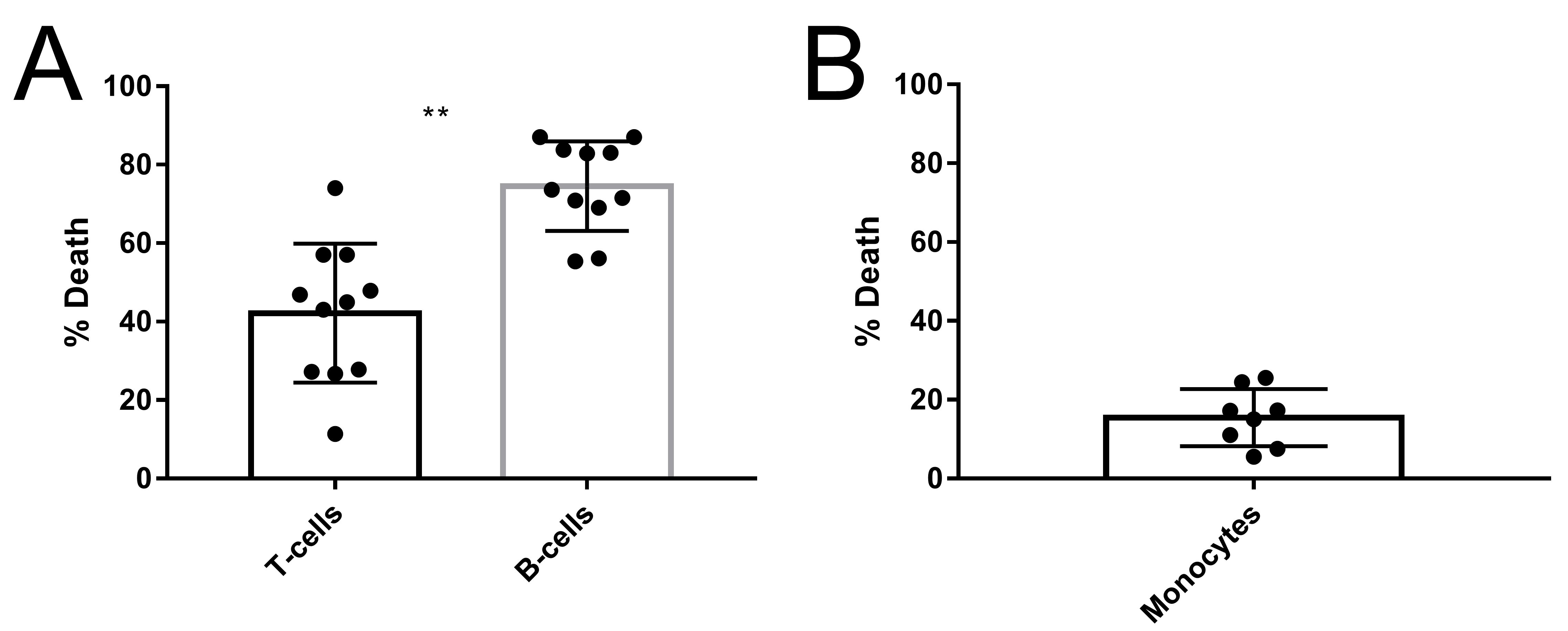

Supplement: S4 Fig — % death is shown for all donors used in the study, at MOI 0.5 TvMSA1132 for 4 hours. B-cell versus T-cell susceptibility was compared using paired (donor-matched), student’s T-test. (TIF) [file pntd.0004913.s004.tif]

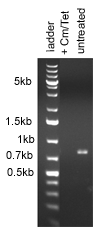

Supplement: S5 Fig — Chloramphenicol/ tetracycline treated (TvMSA1132 Cm/Tet) parasites were analyzed for the presence of bacterial symbionts using universal 16S primers designed to amplify a conserved region of bacterial 16S rDNA. The designed primers amplify a fragment of 834 bp. (Cm: chloramphenicol; Tet: tetracycline) specifically in TvMSA1132 untreated parasites. DNA sequencing of the uncloned, amplified 16S bacterial rDNA fragment from untreated TvMSA1132 was analyzed by BLAST analyses of Genbank. Only one sequence, with 100% homology to 16S region of M. hominis was detected. The sequence matched the following accession numbers with 100% identity: (all strains of M. hominis) CP009652.1, JN935871.1, NR113679.1, NR041881.1, FP236530.1, AF443616.3, AF443617.3, AJ002268.1, AJ002267.1, AJ002266.1, and AJ002265.1. (TIF) [file pntd.0004913.s005.tif]

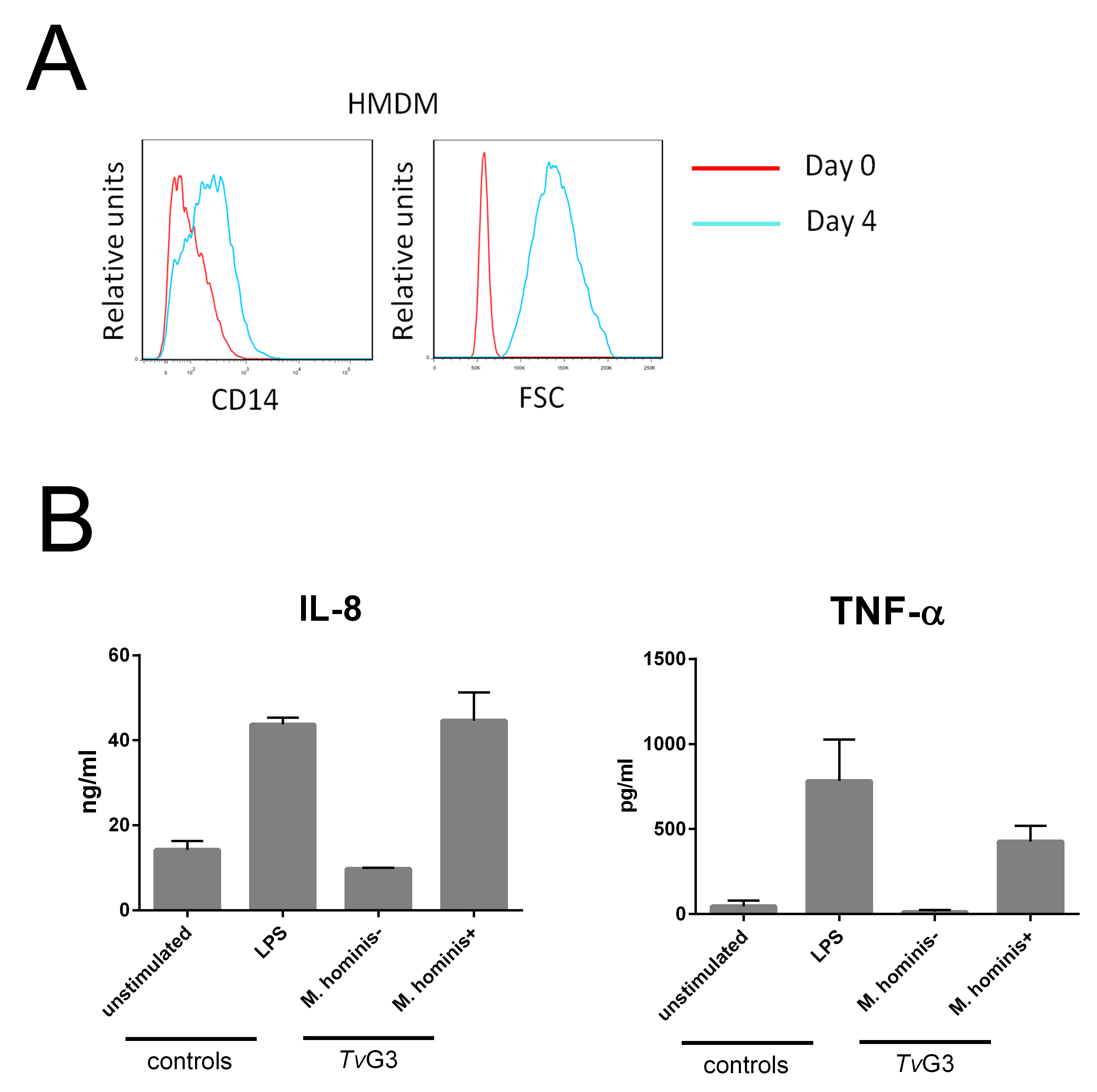

Supplement: S6 Fig — (A) Differentiation of human monocytes to macrophages (HMDM) was verified by the expression of CD14 and the increase in size. (B) HMDM were either unstimulated, treated with LPS, or cultured with heat-inactivated TvG3 for 16 hours. Supernatants were collected and the indicated cytokines were measured using CBA. Data shown are average of triplicate wells with standard deviation, and are representative of 3 donors/ independent experiments. (TIF) [file pntd.0004913.s006.tif]
